# Zero-dc-field rotation-direction-dependent magnetization switching induced by a circularly polarized microwave magnetic field

**DOI:** 10.1038/s41598-017-13770-w

**Published:** 2017-10-23

**Authors:** Hirofumi Suto, Taro Kanao, Tazumi Nagasawa, Koichi Mizushima, Rie Sato

**Affiliations:** Corporate Research and Development Center, Toshiba Corporation, 1, Komukai-Toshiba-cho, Saiwai-ku, Kawasaki, 212-8582 Japan

## Abstract

Magnetization switching of high-anisotropy nanomagnets by a small magnetic field is a key challenge in developing future magnetic nanodevices. In this paper, we experimentally demonstrate magnetization switching of a perpendicularly magnetized nanomagnet induced solely by an in-plane circularly polarized microwave magnetic field. Applying a microwave field with an amplitude below 5% of the anisotropy field induces large ferromagnetic resonance excitation, which results in magnetization switching even in the absence of a dc field. This kind of magnetization switching is induced by a microwave field with a duration of 0.5 ns and is clearly dependent on the rotation direction of the microwave field.

## Introduction

Magnetization switching of nanomagnets is a core technology in magnetic recording. Conventionally, applying a magnetic field larger than the switching field can reverse the magnetization direction. This switching method, however, may not be applicable to future high-density recording devices. This is because nanomagnets require a higher anisotropy to maintain thermal stability as their size becomes smaller to increase recording density, which makes it impossible to generate a sufficiently large magnetic field for switching localized to the nanomagnet. In response to this situation, a switching method based on ferromagnetic resonance (FMR) excitation in a microwave magnetic field has been proposed as a technology for reversing high-anisotropy nanomagnets by a small magnetic field^1–10^. Owing to its resonant nature, a small-amplitude microwave field can induce large FMR excitation, and it has been experimentally reported that the switching field of a nanomagnet can be substantially reduced by applying a microwave field with an amplitude of a few percent of the intrinsic switching field and with a frequency of the order of the FMR frequency^4^. This switching method is called microwave-assisted magnetization switching (MAS) and has been extensively studied as a candidate writing method in next-generation magnetic recording. Furthermore, the use of FMR excitation can provide novel techniques for manipulating magnetization direction. One is layer-selective MAS, which has been explored as a writing method for multilayer recording^7,8^. When a multilayer magnetic structure with each layer having a different FMR frequency is used, switching of only a target layer can be selectively induced by tuning the frequency of the microwave field. The other is spin-wave-assisted magnetization switching. When a coupled structure of a high- and low-anisotropy magnetic material is used, exciting a spin wave in the low-anisotropy material can decrease the switching field of the high-anisotropy material^9^.

In this paper, we experimentally demonstrate magnetization switching of a perpendicularly magnetized nanomagnet induced solely by an in-plane microwave field. The principle behind this switching method is large FMR excitation, which results in magnetization switching without a perpendicular dc magnetic field. In the absence of the dc field, the magnetization is symmetric with respect to the perpendicular direction, and a circularly polarized microwave field is employed to break the symmetry. Because FMR excitation is a precessional motion of the magnetization that rotates in the right-hand screw direction about the equilibrium direction, FMR excitation and subsequent switching occurs only when the microwave field rotates in the same direction. Thus the final magnetization direction is determined by the rotation direction. Switching is induced by a microwave field with a duration of 0.5 ns, showing that this switching method can achieve a high write rate.

The remainder of the paper is organized as follows. After explaining the sample structure and measurement setup, we investigate magnetization switching of a nanomagnet by changing a $z$-direction dc magnetic field (${H}_{z}$), a microwave field amplitude (${H}_{{\rm{rf}}}$), and a microwave field frequency (${f}_{{\rm{rf}}}$), and we show that magnetization switching can be induced solely by a circularly polarized microwave field. We then study the timescale of this kind of magnetization switching and demonstrate successive magnetization manipulation by this switching method. As a related issue, we also discuss the nucleation of a reversed magnetic domain induced by a microwave field. The experimental results are compared with a theoretical study and macrospin simulation.

## Experimental

**Figure 1:**
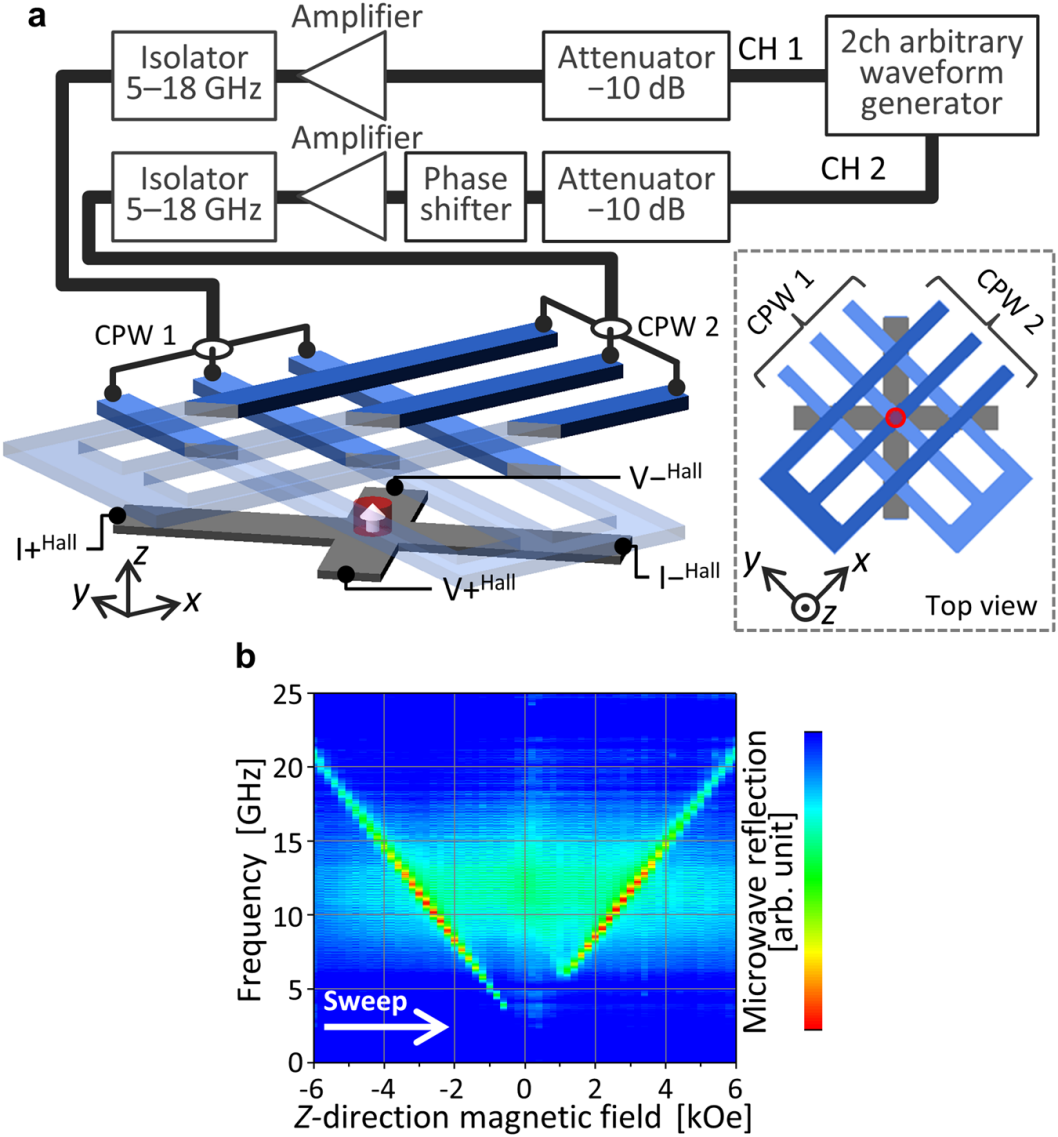
(**a**) Sample structure and experimental setup. (**b**) VNA-FMR spectra versus ${H}_{z}$ obtained for the film sample. The spectra measured at ${H}_{z}=-10$ kOe are subtracted as background.

Figure 1a shows the sample structure and the measurement setup. We study switching of a Co/Pt nanomagnet in ${H}_{z}$ and microwave fields from the two coplanar wave guides (CPWs). The nanomagnet is patterned from a Co/Pt film by electron beam lithography and Ar ion milling. Figure 1b shows the dependence of vector network analyzer (VNA)-FMR spectra on ${H}_{z}$ obtained for the film. The FMR peak disappears at around ${H}_{z}=0$ Oe because reversed magnetic domains are formed. By linear extrapolation, the FMR frequency at ${H}_{z}\,=\,0$ Oe is estimated to be 2.5 GHz, which means that the anisotropy field of the Co/Pt film, including the demagnetizing field, is approximately 900 Oe. Because patterning the film decreases the demagnetizing field, the anisotropy field of the nanomagnets is higher than the film value. To generate microwave fields, two microwave signals are introduced to the CPWs by using the microwave circuit shown in Fig. 1a. The microwave signals are chopped to nanosecond-order pulses to avoid temperature rise of the sample and are emitted repeatedly at a rate of 122 kHz. We mostly discuss magnetization switching in a microwave field using a nanomagnet with a diameter of 50 nm. Afterward, we also discuss reversed-domain nucleation using a 60-nm sample.

**Figure 2:**
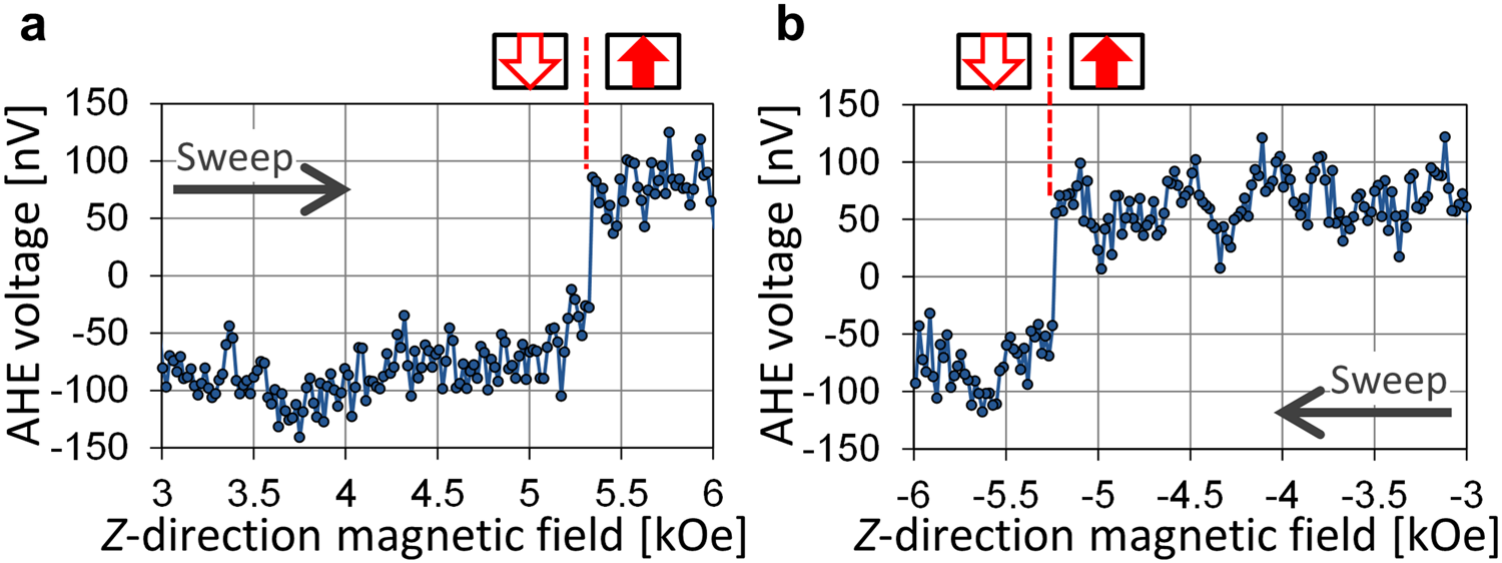
(**a**) and (**b**) AHE voltage versus ${H}_{z}$ for the 50-nm sample. Schematics above the plot depict the magnetization configuration of the nanomagnet. Abrupt changes at ${H}_{z}=\pm 5.3$ kOe correspond to switching of the nanomagnet. Voltage originating from the normal Hall effect of the Ta bottom layer and from the shape of the Hall bar is subtracted as background. This background subtraction is carried out in all AHE measurements.

We confirm that magnetization switching of the 50-nm nanomagnet can be detected by anomalous Hall effect (AHE) measurement (Fig. 2a and b). Here, no microwave field is applied. Abrupt changes in the AHE voltage at ${H}_{z}=\pm 5.3$ kOe correspond to switching of the nanomagnet.

## Results and Discussion

**Figure 3:**
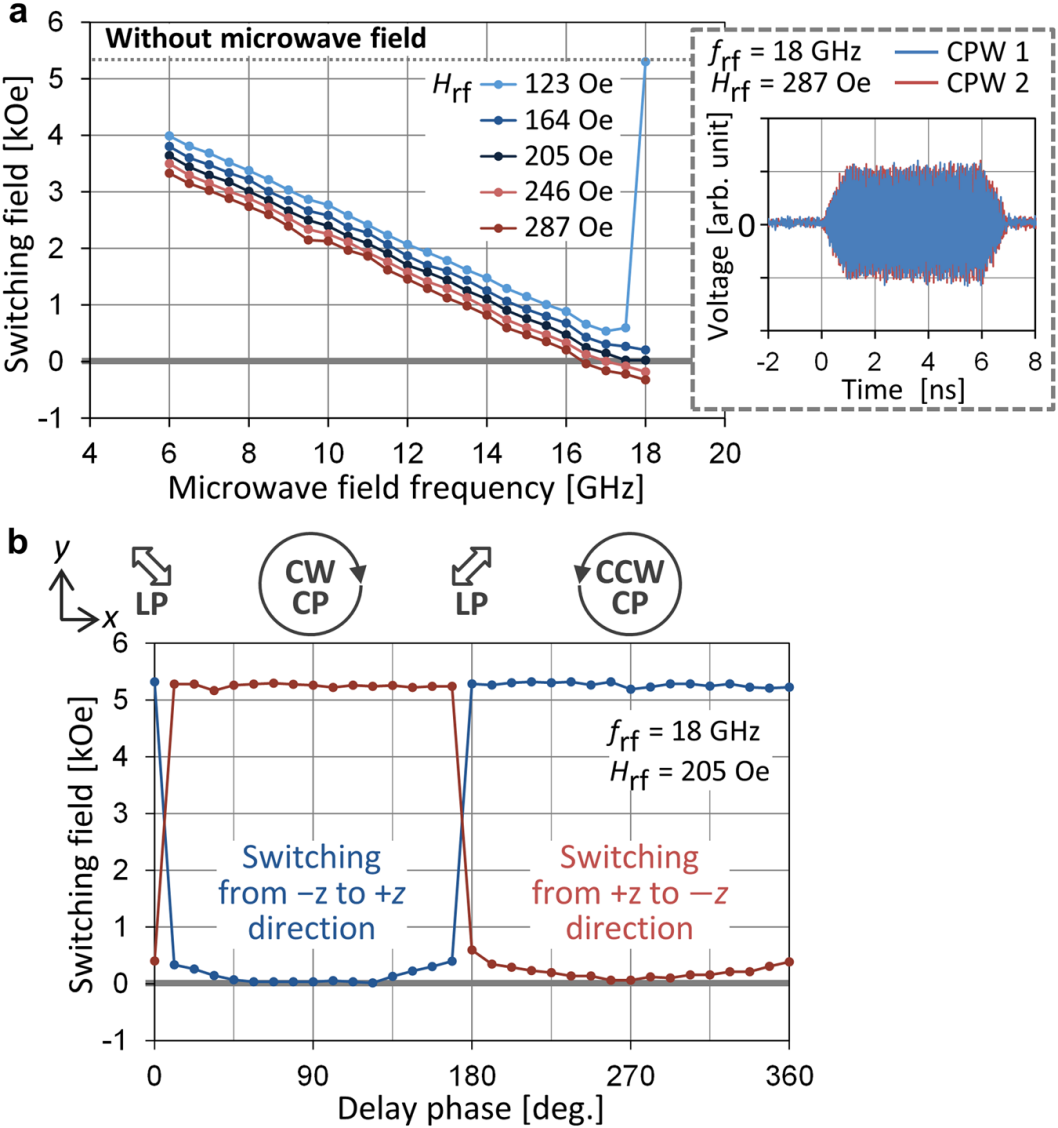
(**a**) ${H}_{{\rm{sw}}}$ versus ${f}_{{\rm{rf}}}$ in the circularly polarized microwave field from CPWs 1 and 2 for several ${H}_{{\rm{rf}}}$ values. The inset shows a waveform of signals with 1-ns rise/fall time and 5-ns plateau time measured at the cable end connected to the CPWs. (**b**) ${H}_{{\rm{sw}}}$ versus delay phase of the signal in CPW 2 with respect to that in CPW 1 for ${f}_{{\rm{rf}}}\,=\,18$ GHz and ${H}_{{\rm{rf}}}=205$ Oe. Schematics at the top depict polarization of the generated microwave field. Two curves correspond to the cases in which the nanomagnet reverses from the $-z$ to $+z$ directions and from the $+z$ to $-z$ directions, respectively.

We first study magnetization switching of the 50-nm nanomagnet by applying a circularly polarized microwave field that rotates clockwise (CW) in the $x$–$y$ plane. This microwave field is generated by adjusting the amplitude of two microwave signals in the CPWs so that the ${H}_{{\rm{rf}}}$ from them becomes the same, and by setting the delay phase between them to 90°. Microwave signals have a 1-ns rise/fall time and a 5-ns plateau time (Fig. 3a inset). When the delay phase is introduced, the signal in CPW 1 rises or falls earlier than that in CPW 2, and ${H}_{{\rm{rf}}}$ from the two CPWs differs during the rise/fall time. This difference in ${H}_{{\rm{rf}}}$ is negligible, however, because the rise/fall time is chosen to be sufficiently longer than the introduced delay time. Figure 3a shows the dependence of a switching $z$-direction dc magnetic field (${H}_{{\rm{sw}}}$) on ${f}_{{\rm{rf}}}$, which is measured as follows: at each ${f}_{{\rm{rf}}}$, the nanomagnet is initialized to the $\mbox{--}z$ direction, and ${H}_{z}$ is increased until switching is detected while a pulsed microwave field is applied repeatedly. Because the microwave field rotates in the same direction as the natural precession of the $\mbox{--}z$-direction magnetization, FMR excitation is induced, which can decrease ${H}_{{\rm{sw}}}$. The ${H}_{{\rm{sw}}}$ curves are obtained by changing ${H}_{{\rm{rf}}}$. They all show a linear decrease with respect to ${f}_{{\rm{rf}}}$ and shift to the lower ${H}_{{\rm{sw}}}$ side as ${H}_{{\rm{rf}}}$ increases. For ${H}_{{\rm{rf}}}\,=\,123$ Oe, ${H}_{{\rm{sw}}}$ increases abruptly to 5.3 kOe at 18 GHz and becomes almost the same as the intrinsic ${H}_{{\rm{sw}}}$ without microwave field. For larger values of ${H}_{{\rm{rf}}}$, no such abrupt increase appears in the frequency range studied here. This switching behavior is similar to that reported in previous MAS experiments. However, when ${H}_{{\rm{rf}}}$ is equal to or greater than 246 Oe, ${H}_{{\rm{sw}}}$ becomes negative at around ${f}_{{\rm{rf}}}\,=\,18$ GHz, which has never been reported. Under this condition, the nanomagnet reverses to the $+z$ direction when ${H}_{z}$ is still applied in the $\mbox{--}z$ direction because the induced large FMR excitation can overcome the switching barrier. This result shows that magnetization switching can be induced solely by a microwave field even in the absence of a perpendicular dc field.

Note that, FMR excitation no longer occurs once the nanomagnet reverses, because the rotation direction of the magnetization also reverses and does not match that of the applied microwave field. Therefore, the final magnetization direction of zero-dc-field switching can be determined by the rotation direction of the microwave field despite the fact that $-z$- and $+z$-direction magnetization is energetically equivalent. This one-way magnetization switching is confirmed in Fig. 3b, which shows the dependence of ${H}_{{\rm{sw}}}$ on the delay phase between the signals in CPWs 1 and 2 for ${f}_{{\rm{rf}}}\,=\,18$ GHz and ${H}_{{\rm{rf}}}\,=\,205$ Oe. Polarization of the microwave field is controlled by the delay phase, and a circularly polarized microwave field rotating CW in the $x$–$y$ plane is generated at 90°, as already used in Fig. 3a. Under this condition, ${H}_{{\rm{sw}}}$ decreases when the nanomagnet reverses from the −$z$ to $+z$ directions, whereas ${H}_{{\rm{sw}}}$ for the opposite switching direction is the same as the intrinsic ${H}_{{\rm{sw}}}$. In contrast, ${H}_{{\rm{sw}}}$ for switching from the $+z$ to $-z$ directions decreases at 270° where a microwave field rotates counterclockwise (CCW) in the $x$–$y$ plane.

**Figure 4:**
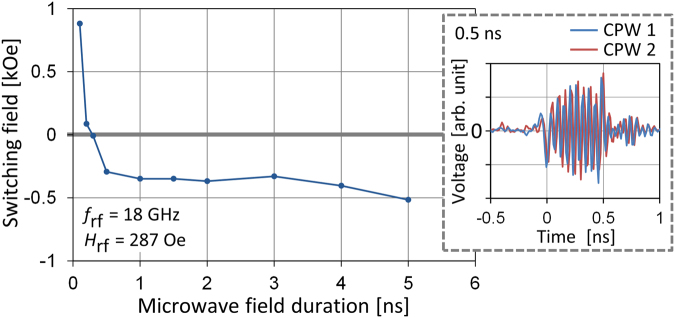
${H}_{{\rm{sw}}}$ versus microwave field duration in the circularly polarized microwave field from CPWs 1 and 2 for ${f}_{{\rm{rf}}}\,=\,18$ GHz and ${H}_{{\rm{rf}}}=287$ Oe. The inset shows a waveform of signals with a zero rise/fall time and a 0.5-ns plateau time measured at the cable end connected to the CPWs.

Thus far, a microwave field with a 1-ns rise/fall time and a 5-ns plateau time was employed to show that zero-dc-field magnetization switching can be induced by a circularly polarized microwave field, and that the final magnetization direction can be determined by the rotation direction of the microwave field. Now, we investigate the timescale of this kind of magnetization switching by setting the rise/fall time to zero and changing the plateau time. The timescale is of practical importance because it affects the write rate. The inset in Fig. 4 shows a waveform of signals with a duration of 0.5 ns. It is confirmed that the signal amplitude is almost the same as for signals with a 1-ns rise/fall time and a 5-ns plateau time (Fig. 3a inset), though it fluctuates because of the sudden rise and fall of the signals. Figure 4 shows the dependence of ${H}_{{\rm{sw}}}$ on the microwave field duration for ${f}_{{\rm{rf}}}\,=\,18$ GHz and ${H}_{{\rm{rf}}}\,=\,287$ Oe. As the microwave field duration increases from 0.1 ns to 0.5 ns, ${H}_{{\rm{sw}}}$ gradually decreases, and when the microwave field duration exceeds 0.5 ns, ${H}_{{\rm{sw}}}$ becomes negative and nearly constant, which is explained as follows. When the duration is shorter than 0.5 ns, the FMR excitation is still developing. Because larger FMR excitation leads to smaller ${H}_{{\rm{sw}}}$, ${H}_{{\rm{sw}}}$ gradually decreases. FMR excitation then saturates at 0.5 ns, and longer application of the microwave field has little effect on ${H}_{{\rm{sw}}}$. The fact that ${H}_{{\rm{sw}}}$ becomes negative at 0.5 ns shows that zero-dc-field magnetization switching can be induced by a microwave field with a duration of 0.5 ns.

For the microwave field duration from 0.5 to 5 ns, ${H}_{{\rm{sw}}}$ almost agrees with the corresponding ${H}_{{\rm{sw}}}$ (${f}_{{\rm{rf}}}\,=\,18$ GHz and ${H}_{{\rm{rf}}}\,=\,287$ Oe) in Fig. 3a. Considering that the signal employed in Fig. 3a has 1-ns rise/fall time and little amplitude fluctuation (Fig. 3a inset), the agreement in ${H}_{{\rm{sw}}}$ indicates that the amplitude fluctuation arising from the sudden rise and fall does not play a crucial role in the 0.5-ns magnetization switching.

**Figure 5:**
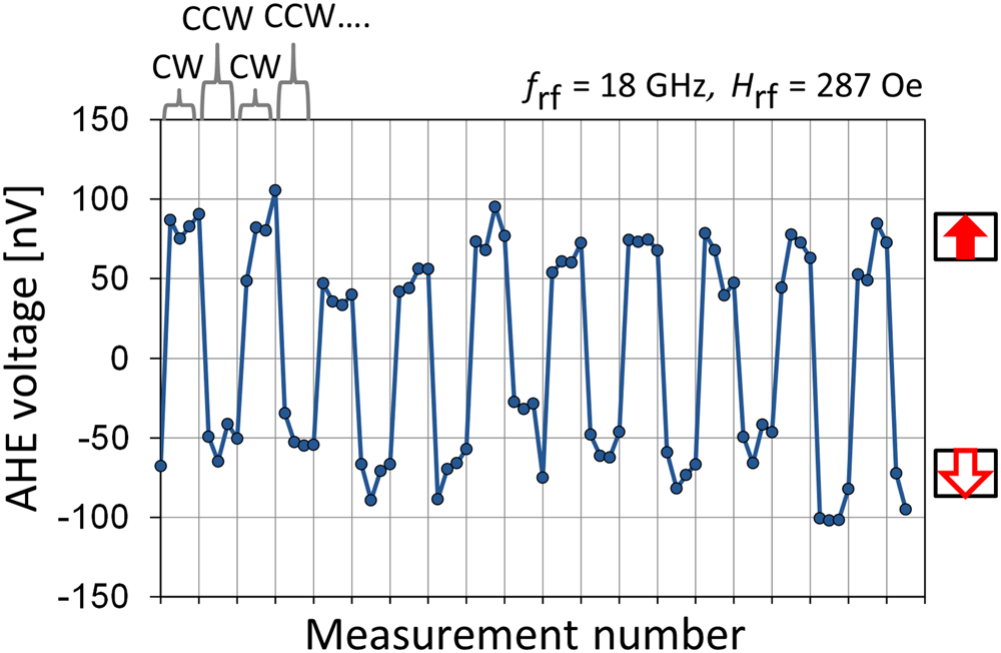
AHE voltage measured by applying the CP microwave field from CPWs 1 and 2 for ${f}_{{\rm{rf}}}\,=\,18$ GHz, ${H}_{{\rm{rf}}}=287$ Oe, and a duration of 0.5 ns. The rotation direction of the microwave field is reversed every four AHE measurements.

We next demonstrate successive magnetization manipulation of the nanomagnet by means of zero-dc-field rotation-direction-dependent magnetization switching. Figure 5 shows AHE voltage at ${H}_{z}\,=\,0$ Oe obtained by reversing the rotation direction of the microwave field every four AHE measurements, while a microwave field with ${f}_{{\rm{rf}}}=18$ GHz, ${H}_{{\rm{rf}}}\,=\,287$ Oe, and a duration of 0.5 ns is applied repeatedly. The nanomagnet is first initialized to the $\mbox{--}z$ direction, and during the next four AHE measurements, a CW microwave field is applied by setting the delay phase to 90°. The AHE signal increases abruptly, showing that the nanomagnet reverses to the $+z$ direction. Similarly, during the next four AHE measurements, a CCW microwave field is applied by setting the delay phase to 270°, and the nanomagnet reverses to the $-z$ direction. It is seen that the AHE voltage changes in accordance with changes in the rotation direction of the microwave field, showing that the magnetization direction is manipulated by the rotation direction of the microwave field. The AHE voltage fluctuates on the order of 50 nV. This fluctuation is due to noise in the AHE measurement—as also seen in Fig. 2a and b—and is not due to the formation of a reversed magnetic domain, which is expected to result in an intermediate AHE voltage. We explain this issue of magnetic domain configuration by referring to the results from a slightly larger sample in the following paragraph.

**Figure 6:**
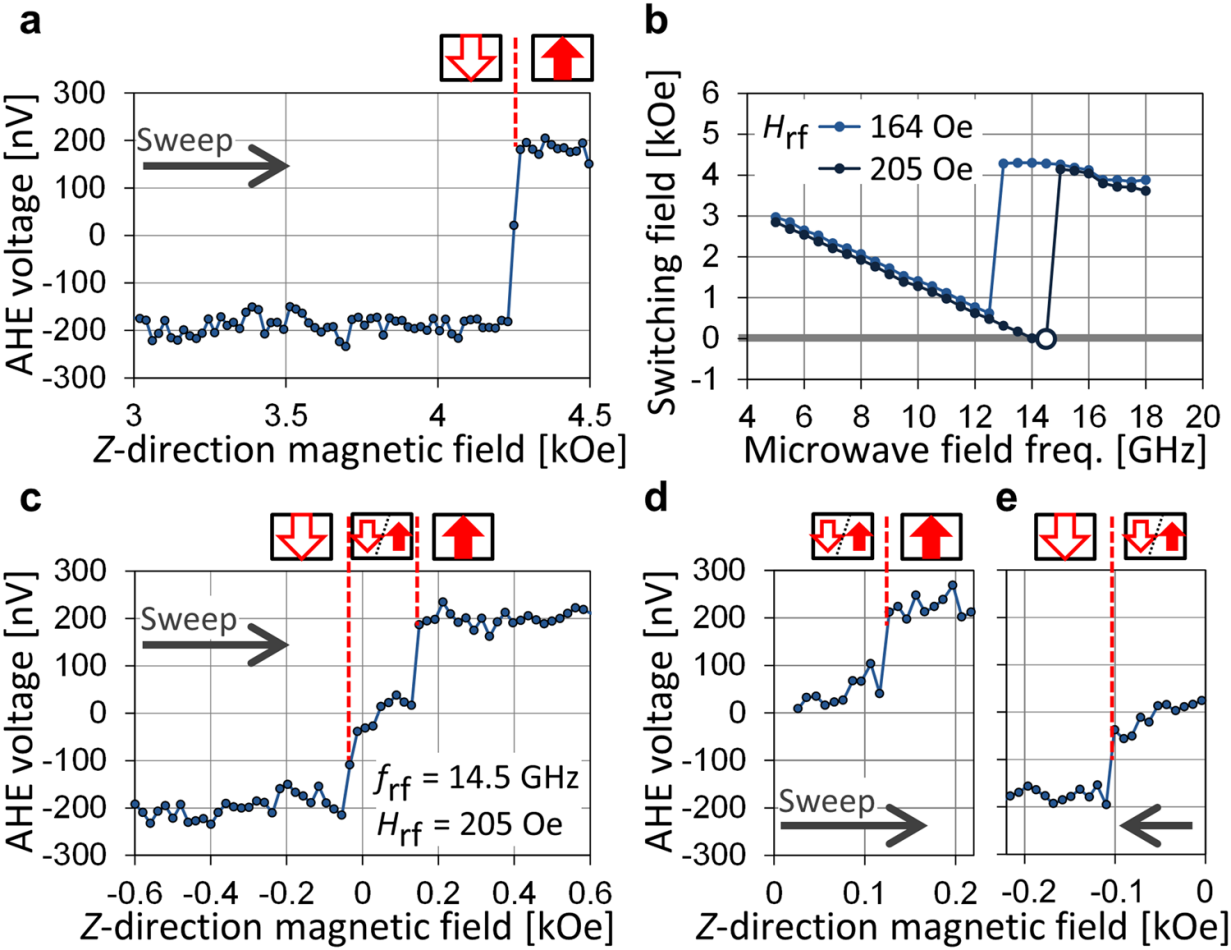
(**a**) AHE voltage versus ${H}_{z}$ for the 60-nm sample. Abrupt changes at ${H}_{z}\,=$ +4.3 kOe correspond to switching of the nanomagnet. (**b**) ${H}_{{\rm{sw}}}$ versus ${f}_{{\rm{rf}}}$ in the circularly polarized microwave field from CPWs 1 and 2. The open circle indicates that the reversed domain is formed. (**c**) AHE voltage versus ${H}_{z}$ in the CP microwave field from CPWs 1 and 2 for ${f}_{{\rm{rf}}}=14.5$ GHz and ${H}_{{\rm{rf}}}=205$ Oe. (**d**) and (**e**) AHE voltage versus ${H}_{z}$ obtained by sweeping ${H}_{z}$ in the upward and downward direction after the reversed domain is formed. The reversed domain is formed by applying the microwave field with the same parameters as in (**c**), and during the ${H}_{z}$ sweep, the microwave field is terminated.

Figure 6a shows the dependence of AHE voltage on ${H}_{z}$ for a sample with a diameter of 60 nm. Here, no microwave field is applied. An abrupt change in the AHE corresponding to switching appears at ${H}_{z}=+4.3$ kOe. The AHE voltage amplitude is larger than that for the 50-nm sample because of the size, and ${H}_{{\rm{sw}}}$ is lower than that for the 50-nm sample because the larger sample has a larger demagnetizing field and thus a smaller anisotropy field. Figure 6b shows the ${f}_{{\rm{rf}}}$ dependence of ${H}_{{\rm{sw}}}$. Similar to the 50-nm sample, ${H}_{{\rm{sw}}}$ decreases as ${f}_{{\rm{rf}}}$ increases. However, the reversed magnetic domain is formed at ${f}_{{\rm{rf}}}=14.5$ GHz, as indicated by the open circle. The corresponding ${H}_{z}$ dependence of AHE voltage is shown in Fig. 6c. An abrupt change in the AHE voltage at ${H}_{z}\,=\,50$ Oe is due to formation of the reversed magnetic domain, and an intermediate AHE voltage appears. This result shows that part of the nanomagnet reverses to the $+z$ direction owing to the large FMR excitation despite ${H}_{z}$ being applied in the $\mbox{--}z$ direction. Then, magnetization of the nanomagnet saturates in the $+z$ direction at ${H}_{z}=+150$ Oe, where the AHE voltage increases again. Once the reversed magnetic domain is formed, applying ${H}_{z}$ of approximately $\pm 100$ Oe is necessary to make the magnetization uniform in the $+z$ or $-z$ direction, as shown in Fig. 6d and e. Applying a microwave field with the same ${f}_{{\rm{rf}}}$ as the one that forms the reversed domain cannot change the magnetic configuration because the FMR excitation of the domain configuration differs drastically from that of the uniform configuration. Therefore, the fact that switching occurs repeatedly and without fail for the 50-nm sample (Fig. 5) evidences that a reversed magnetic domain is not formed, and that complete switching of the nanomagnet always occurs. The comparison of the samples of different sizes indicates that nanomagnets need to be small enough to prevent domain configuration when zero-dc-field switching by a microwave field is carried out.

We next comment on the comparison with a theoretical study based on the zero-temperature macrospin model. Taniguchi *et al*. have shown that zero-dc-field switching is induced when a microwave field with ${H}_{{\rm{rf}}}$ larger than 15% of the anisotropy field is applied^10^. When we employ the intrinsic ${H}_{{\rm{sw}}}$ of 5.6 kOe as an anisotropy field for a rough estimation, ${H}_{{\rm{rf}}}$ in our experiment is at most 5% of the anisotropy field. The disagreement between ${H}_{{\rm{rf}}}$ values in the experiment and the theory is partly explained by the fact that zero-dc-field switching in our experiment is thermally activated, whereas the theoretical value is required for magnetization switching at zero temperature. In addition, previous MAS studies on a nanomagnet with a diameter of a few tens to 100 nm have reported that MAS effect is enhanced in comparison with the macrospin theory because spatially non-uniform magnetization excitation occurs in the nanomagnet. This magnetization excitation leads to nucleation of a reversed domain, and magnetization switching occurs as this domain expands. This nucleation-type magnetization switching is supported by the fact that the dot diameter of our samples is larger than the exchange length of the material^1^, and by the experimental result that the reversed domain actually appears in the 60-nm sample. In the 50-nm sample, the nucleated reversed domain spontaneously expands to the edge and leads to complete switching of the nanomagnet, whereas in the 60-nm sample, the reversed domain can exist when ${H}_{z}$ is in the range of $\pm 100$ Oe.

**Figure 7:**
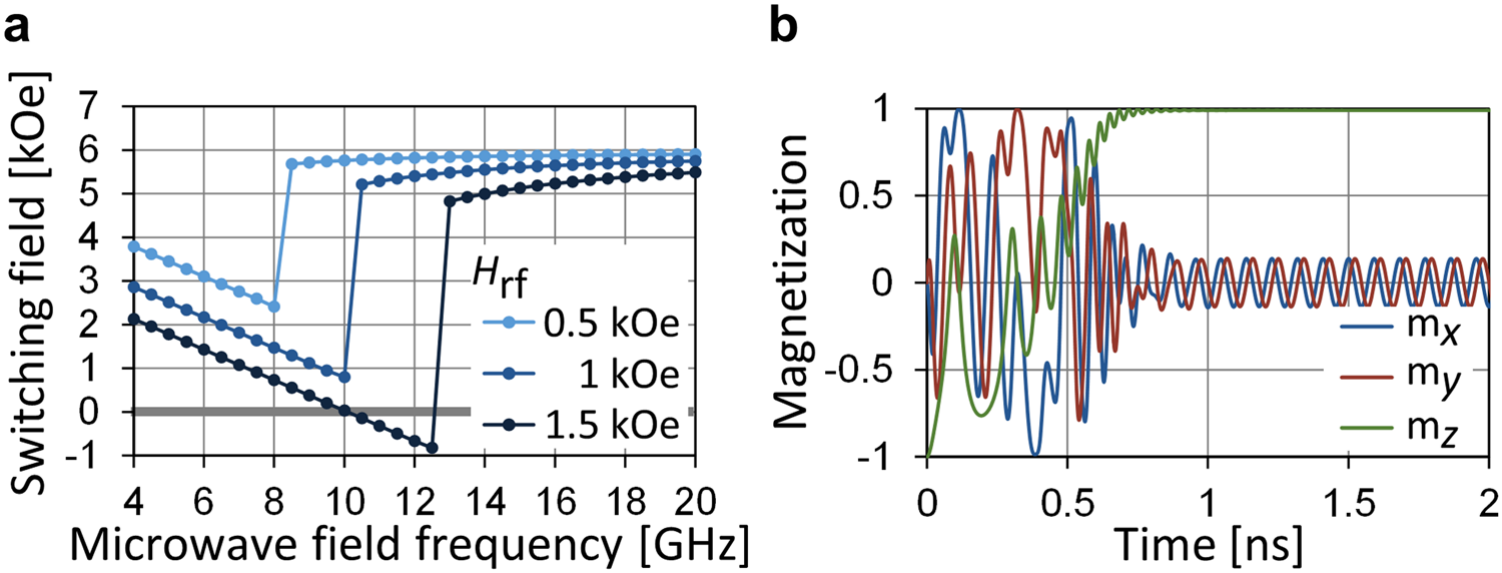
(**a**) ${H}_{{\rm{sw}}}$ versus ${f}_{{\rm{rf}}}$ obtained by macrospin simulation. (**b**) Time evolution of $x$-, $y$-, and $z$-components of the magnetization direction for ${f}_{{\rm{rf}}}\,=\,11$ GHz, ${H}_{{\rm{rf}}}=1.5$ kOe, and ${H}_{z}=0$ Oe.

Figure 7a shows the result of zero-temperature macrospin simulation. Similar to the experimental results, ${H}_{{\rm{sw}}}$ decreases with increasing ${f}_{{\rm{rf}}}$ and becomes negative when ${H}_{{\rm{rf}}}$ is sufficiently large. As already discussed, ${H}_{{\rm{rf}}}$ required for zero-dc-field switching is larger in the macrospin description than in the experimental result. Figure 7b shows time evolution of the magnetization direction for zero-dc-field switching. Switching is completed approximately in 0.5 ns, which is on the same timescale as the experimental results.

In the experiments, a circularly polarized microwave field is generated by the two crossing CPWs. A circularly polarized microwave field can also be generated by a spin-torque oscillator (STO) having a pinned layer with perpendicular magnetization^11–13^ In this kind of STO, the free-layer magnetization rotates about the perpendicular direction with a large cone angle and generates a stray field rotating about the perpendicular direction. The STO is practically advantageous because it generates a microwave field simply by applying a dc current. In addition, because the STO can be as small as 30 nm in diameter, the generated microwave can manipulate magnetization in the nanoscale. By reversing the pinned-layer magnetization direction or the current direction, the rotation direction of the microwave field can be controlled, which is crucial to determining the final magnetization direction in the zero-dc-field switching.

In summary, we have demonstrated zero-dc-field magnetization switching of a perpendicularly magnetized nanomagnet induced by a circularly polarized microwave field. When the nanomagnet is sufficiently small to prevent magnetic domain configuration, a microwave field with an amplitude approximately 5% of the intrinsic switching field can reverse the magnetization direction. The final magnetization direction is determined by the rotation direction of the microwave field, and switching is induced by a microwave field with a duration of 0.5 ns. These findings show that this method is promising technology for switching high-anisotropy nanomagnets with a low-amplitude magnetic field in the subnanosecond timescale.

## Methods

### Sample fabrication

A Ta bottom layer and perpendicularly magnetized Co/Pt film is deposited on a sapphire substrate by using a magnetron sputtering system. The film structure consists of the following layers from bottom to top: Ta 200/Pt 50/(Co 14.3/Pt 6) ×5. Thicknesses are given in angstroms. The Co/Pt film is then patterned into a nanomagnet by electron beam lithography and Ar ion milling. The Ta bottom layer is patterned into a Hall cross to detect switching of the nanomagnet by AHE measurement. On the nanomagnet, two insulating layers and two layers of CPWs are alternately fabricated. The CPWs consist of a 1-μm-wide signal line and two 2-μm-wide ground lines with 2-μm gaps between them, which make the characteristic impedance approximately 50 Ω. The signal lines of the two CPWs cross at a right angle above the nanomagnet and the CPWs are short-terminated after passing above the nanomagnet.

### Measurement Procedure

To generate the microwave fields, two microwave signals are introduced to the CPWs by using the microwave circuit shown in Fig. 1a. In the circuit, the attenuators trim the amplitude of the microwave signal from the arbitrary waveform generator, and the isolators with a 5 GHz−18 GHz bandwidth protects the amplifier from the reflected signal. Polarization of the microwave field is controlled by the delay between microwave signals, which is introduced by the sample clock delay of the arbitrary waveform generator and by the phase shifter. We estimate ${H}_{{\rm{rf}}}$ by assuming a uniform current in the signal line and using the Biot-Savart formula. The microwave signals are chopped to nanosecond-order pulses to avoid temperature rise of the sample and are emitted repeatedly at a rate of 122 kHz. During AHE measurements, which take approximately 0.3 s for averaging, ${H}_{z}$ is constant. In ${H}_{z}$ sweep measurements, ${H}_{z}$ is changed in steps of 10 Oe during the intervals between the AHE measurements. All measurements are carried out at room temperature.

### Macrospin simulation

Magnetization dynamics is calculated by using the Landau–Lifshitz–Gilbert equation. The saturation magnetization (${M}_{{\rm{s}}}$) is 1000 emu/cm^3^, and the damping constant is 0.1. The perpendicular anisotropy field is set to $4{\rm{\pi }}{M}_{{\rm{s}}}+900$ Oe, based on the VNA-FMR measurement (Fig. 1b). The demagnetizing field is calculated by assuming a cylindrical magnet with a diameter of 50 nm and a thickness of 10 nm. In Fig. 7a, at each ${f}_{{\rm{rf}}}$, the magnetization is initialized to the $\mbox{--}z$ direction, and ${H}_{z}$ is increased from −1 kOe to 9 kOe over a 10-μs period while a microwave field rotating CW in the $x$–$y$ plane is applied continuously. Here, ${H}_{z}$ is tilted 2° from the $z$ direction. In Fig. 7b, the magnetization is initialized to the $\mbox{--}z$ direction, and only a microwave field rotating CW in the $x$–$y$ plane is applied.

### Data Availability

The datasets generated during and/or analysed during the current study are available from the corresponding author on reasonable request.
